# Metagenomic 16S rDNA amplicon data of microbial diversity of *Cimex hemipterus* (F.) (Hemiptera: Cimicidae) treated with insect growth regulators (IGR)

**DOI:** 10.1016/j.dib.2023.109024

**Published:** 2023-03-01

**Authors:** NurHidayah Taibukahn, Abdul Hafiz Ab Majid

**Affiliations:** a Household and Structural Urban Entomology Laboratory, Vector Control Research Unit, School of Biological Sciences, Universiti Sains Malaysia, 11800 Minden, Pulau Pinang, Malaysia; b Centre for Insect Systematics (CIS), Faculty of Science and Technology, Universiti Kebangsaan Malaysia (UKM), Bangi 43600, Malaysia

**Keywords:** Tropical bed bug, *Cimex hemipterus*, *Metagenomics*, *IGR treatment*, *Microbial diversity*

## Abstract

The metagenomics dataset presented here is based on bacterial 16S rDNA gene amplicons of DNA extracted from tropical bed bugs (*Cimex hemipterus*). Amplicon-based sequencing was performed using the Illumina MiSeq platform, and the raw sequence data were analyzed using QIIME (version 2022.8.3). The metagenome sequence comprised ten samples that include C1 (133 511bps), C2 (108 920bps), CH1 (106 562bps), CH2 (101 778bps), P1 (103 618bps), P2 (133 258bps), T1 (113 558bps), T2 (133 952bps), TM1 (125 335bps), and TM2 (118 345bps). The sequence data is readily accessible at the NCBI SRA under bio project PRJNA918835. The most abundant microbial community present in the *C. hemipterus* is the Proteobacteria, with more than 99% of the abundance.

Specification table

**Table:** 

Subject	Microbiology: Microbiome

Specific subject area	A metagenomic study on the microbiota of tropical bed bugs, *Cimex hemipterus*, treated with insect growth regulators (IGR)*.*
Type of data	Table, figures, and 16S rDNA Illumina sequences.
How the data were acquired	16S rDNA Illumina sequencing followed by community metagenome analysis.
Data format	Raw: FASTQ file.
Description of data collection	The HiYield TM Genomic DNA isolation kit (Real Biotech Corporation, Taiwan) was used to recover the microbial DNA from fully-fed tropical bed bugs that had been crushed. Illumina MiSeq platform was used to carry out 16S v3-v4 amplicon metagenomics sequencing.
Data source location	Bed bugs were collected from residential houses during the visual inspection using a flashlight and forceps. The GPS coordinates are: 5.41153N 100.33367E, 5.32937N 100.27653E, 5.39924N 100.28471E, 5.41333N 100.32989E, and 5.36743N 100.27439E.
Data accessibility	^1^Repository name: NCBI SRA Data identification number: PRJNA918835 Direct URL to data: https://www.ncbi.nlm.nih.gov/bioproject/PRJNA918835/

## Value of the data

• The metagenomics data offer comprehensive taxonomic profiles of microbial abundance and diversity in *Cimex hemipterus* treated with insect growth regulators.
• The dataset discloses information regarding the effect of insect growth regulators on the microbial community of *C.hemipterus*.
• The data also provides knowledge for examining the variation of microbiome influence that may contribute to a pest management approach that encourages the exploration of novel targets of chemical, genetic, and biological control for the *Cimicidae* family.

## 1 Objective

*Cimicidae* families are among the significant dilemmas of household pest management strategies since the bed bug infestation rate has failed to be fully curbed as bed bugs have the resistance capability that prolongs their survival and allows positive breeding patterns. Thus, insect growth regulators (IGR), a current key strategy to quell bed bug infestation, have been implemented. IGRs are best known as the juvenile hormone analog that hampers embryogenesis and reproduction, while chitin synthesis inhibitors impede the formation of the exoskeleton. However, IGRs are beginning to show the odds of resistance as bed bug infestations are reclaiming their title as a nuisance pest. To understand the potential cause of the resistance, it is crucial to consider the microbiome interaction with the IGR application. Hence, this study determines the microbial community of treated and untreated *Cimex hemipterus* exposed to IGRs using the 16S rDNA metagenomic analysis. Further research is required to determine the presence of substantial or insignificant differences in the microbial diversity between the control (untreated) and treated tropical bed bugs.

## 2 Data description

The presented dataset consists of bacterial metagenomic sequencing of control (untreated) and IGR-treated tropical bed bugs using an Illumina Miseq sequencer, which produces sequences with average reads from samples. A total of ten samples, comprised of two untreated control samples (C1 and C2) and eight IGR-treated samples (CH1, CH2, P1, P2, T1, T2, TM1, and TM2), are presented in Table 1. Samples with similar alphabets represent two biological replicates of each sample from similar sites and strains. Two similar control samples served as negative controls and were not treated with growth regulators.

**Table 1:** The number of sequences, base pairs, and the average length of *Cimex hemipterus* samples.

Sample	Sequences	Bases (bp)	Average length (bp)
C1	133,511	54,664,740	409.44
C2	108,920	44,170,945	405.54
CH1	106,562	43,279,718	406.15
CH2	101,778	41,549,419	408.24
P1	103,618	44,158,666	426.17
P2	133,258	56,626,176	424.94
T1	113,558	46,267,932	407.44
T2	133,925	54,783,838	409.06
TM1	125,335	50,909,020	406.18
TM2	118,345	48,018,195	405.75

**Fig. 1:**
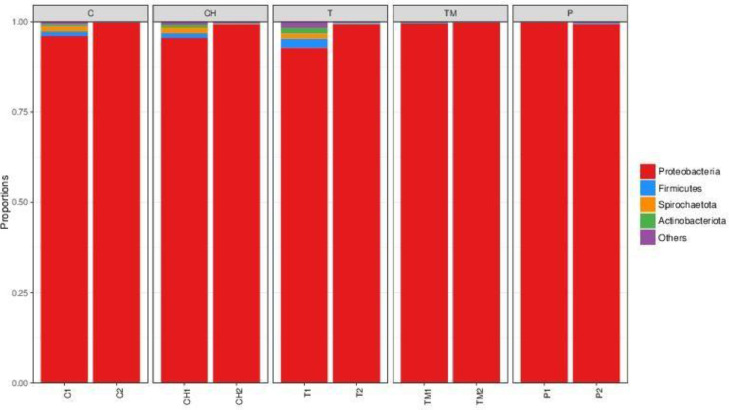
Stacked bar chart showing the taxonomic abundance at the phylum level of microbial communities between untreated (C1 and C2) and treated (CH1, CH2, T1, T2, TM1, TM2, P1, and P2) samples of *Cimex hemipterus*. The X-axis represents two biological replicates of pooled samples for each growth regulator, while the Y-axis is the taxon abundance. Phyla, which received less than 1%, were clubbed and assigned to 'Others.'

The community analysis reveals the Proteobacteria family as the predominant microbial phylum, with more than 98% sequences for treated and untreated samples. The families were primarily divided into two genera, namely Wolbachia and Pectobacterium. Spirochaetota has 1%-marking reads, while the other eight different phyla of bacteria, including Actinobacteriota, Bacteroidota, Desulfobacterota, Fibrobacterota, Firmicutes, Unclassified, Rs-K70 termite group, and Synergistota, have read rates of less than 1% (Figure 1).

## 3 Experimental design, material, and methods

The metagenomic analysis was carried out on tropical bed bugs, *C.hemipterus,* collected from residential areas in Penang Island to validate their bacterial composition. Collected samples were treated with two classes of IGRs, namely the juvenile hormone analog and the chitin synthesis inhibitor [1]. Once the treated bed bug reached mortality, the samples were pooled for DNA extraction, using five bed bugs per triplicate. The samples were sterilized twice, once with 75% ethanol for 30 seconds and once with sterile distilled water for a minute, to remove the presence of external contaminants before molecular analysis. Tropical bed bugs were homogenized, and the genomic DNA was extracted using the HiYield™ Genomic DNA isolation kit (Real Biotech Corporation, Taiwan) according to the manufacturer's protocols with minimal modifications, including using 40µl Proteinase K instead of 20 µl and reducing the incubation period from three hours to one hour (Ashigar & Ab Majid, 2020 [2]). Polymerase chain reaction (PCR) amplification was performed on the V3-V4 region of the bacteria from the extracted gDNA samples using PCR protocols (95 °C for 2 min, followed by 25 cycles at 95 °C for 30 s, 55 °C for 30 s, and 72 °C for 30 s, and a final extension at 72 °C for 5 min) (Ashigar & Ab Majid, 2021 [3]).

**Fig. 2:**
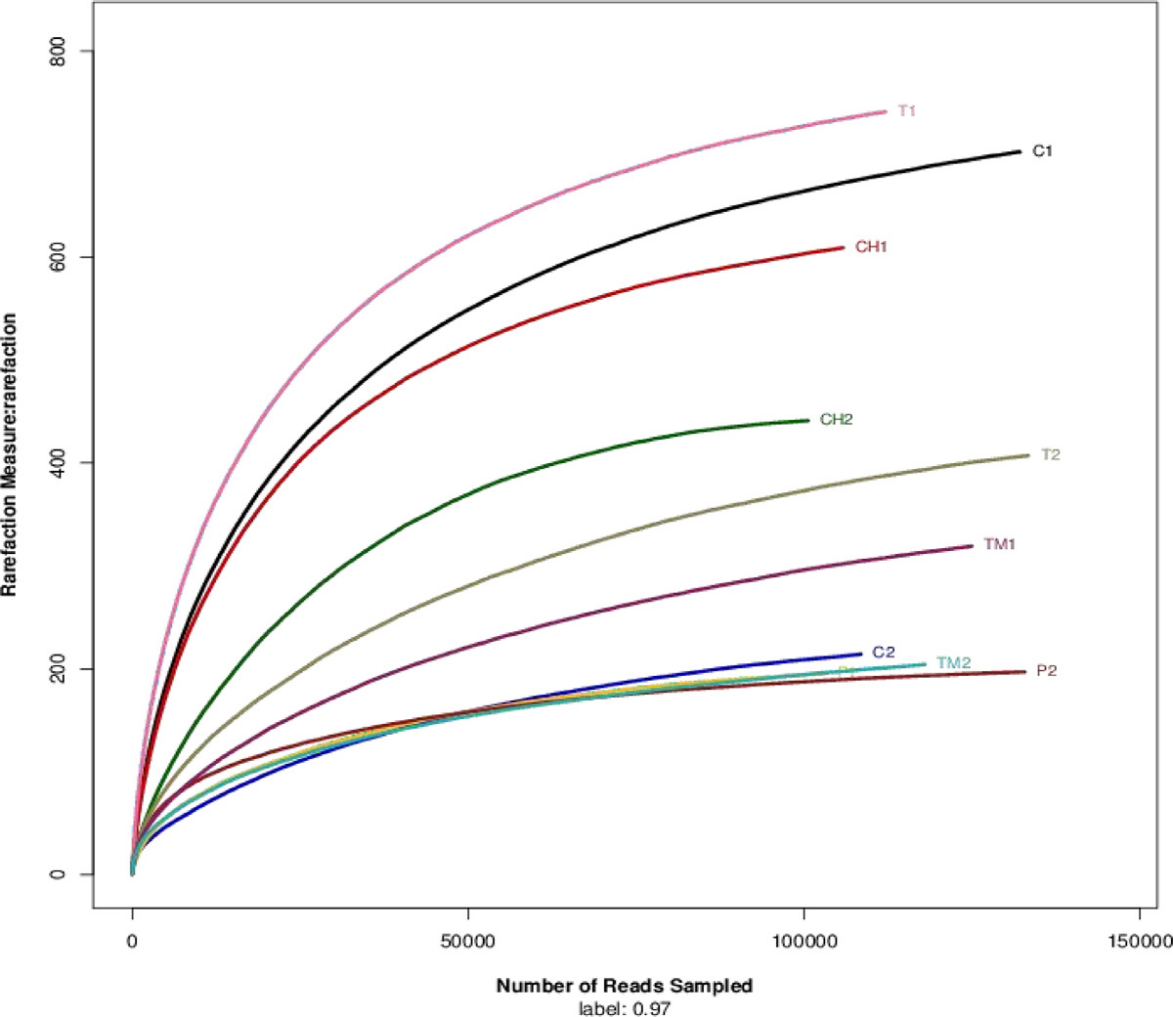
The rarefaction curves exhibit the species richness calculation of the microbiome of *C. hemipterus* samples.

Based on the standard protocol of the Illumina Miseq platform, the amplicon library was constructed, and QIIME (version 2022.8.3) was used to analyze the raw sequences. The reads were trimmed using Trimmomatic software, which naturally discarded reads less than 50 bp and gave the reads an average quality score of below 20. Using FLASH (Fast Length Adjustment of Short Reads), paired reads were integrated into a single read based on an overlapping relationship and reassembled overlapping sequences that were longer than 10 bp while deleting unassembled reads (Lim & Ab Majid, 2020 [5]). Operational Taxonomic Units (OTU) were assigned to a taxonomy using the Ribosomal Database Project (RDP) (Gu et al., 2013 [4]). The OTU employed UPARSE software to cluster the data sets based on a 97% similarity cut-off and UCHIME software to identify the chimeric sequences due to the vast number of reads analyzed (López-García et al., 2018 [6]; Xie et al., 2016 [8]). The RDP Classifier was used to examine the taxonomy of the 16S rRNA gene sequences against the SILVA 16S rRNA database with a confidence level of 0.7. By using the mothur and R software, the sequence coverage of each sample (C1, C2, CH1, CH2, P1, P2, T1, T2, and TM1) was assessed (Fig. 2) (Wu et al., 2019 [7]).

## Ethics statements

The study was conducted according to the guidelines of the Declaration of Helsinki, and approved by the Universiti Sains Malaysia Research Ethics Committee (Human) JEPeM, Code: USM/JEPeM/19120868.

## CRediT authorship contribution statement

**NurHidayah Taibukahn:** Methodology, Investigation, Data curation, Formal analysis, Writing – original draft, Writing – review & editing. **Abdul Hafiz Ab Majid:** Conceptualization, Supervision, Project administration, Resources, Funding acquisition, Writing – review & editing.

## Declaration of Competing Interest

The authors declare that they have no known competing financial interests or personal relationships that could have appeared to influence the work reported in this paper.

## Data Availability

Metagenomic data of microbial diversity of Cimex hemipterus Raw sequence reads (Original data) (NCBI)
